# Evaluating the Contamination by Indoor Dust in Dubai

**DOI:** 10.3390/toxics11110933

**Published:** 2023-11-17

**Authors:** Yousef Nazzal, Alina Bărbulescu, Manish Sharma, Fares Howari, Muhammad Naseem

**Affiliations:** 1 College of Natural and Health Sciences, Zayed University, Abu Dhabi P.O. Box 144534, United Arab Emirates; yousef.nazzal@zu.ac.ae (Y.N.); manish.sharma@zu.ac.ae (M.S.); muhammad.naseem@zu.ac.ae (M.N.); 2 Department of Civil Engineering, Transilvania University of Brașov, 5 Turnului Str., 900152 Brasov, Romania; 3 College of Arts and Sciences, Fort Valley State University, Fort Valley, GA 31030, USA; Fares.Howari@fvsu.edu

**Keywords:** contamination, dust, clustering, pollution index

## Abstract

Nowadays, people spend most of their time indoors. Despite constantly cleaning these spaces, dust apparition cannot be avoided. Since dust can contain chemical elements that negatively impact people’s health, we propose the analysis of the metals from the indoor dust component collected in different locations in Dubai, UAE. Multivariate statistics (correlation matrix, clustering) and quality indicators (QI)—*I_geo_*, PI, EF, PLI, Nemerow—were used to assess the contamination level with different metals in the dust. We proposed two new QIs (CPI and AQI) and compared the results with those provided by the most used indices—PLI and Nemerow. It is shown that high concentrations of some elements (Ca in this case) can significantly increase the values of the Nemerow index, CPI, and AQI. In contrast, the existence of low concentrations leads to the decrement of the PLI.

## 1. Introduction

Indoor dust is the settled particulate matter (PM) found on carpets, floors, surfaces, and other objects in an indoor space. Among other pollutants from indoor dust, heavy metals require extensive research due to their non-degradable properties, high toxicity, and adverse effects on humans [1,2]. The United States Environmental Protection Agency (USEPA) has raised the alarm about indoor air quality, considering it a significant concern because it tends to be more polluted than outdoor air. This concern has grown because people spend a significant portion of their time indoors, encompassing homes, workplaces, schools, public spaces like shops, restaurants, and vehicles, amounting to up to 90% of their daily activities [3]. Children, who spend most of their day at home, are particularly vulnerable to environmental stressors because their breathing zone is close to the floor, where residential dust tends to collect, exposing them to potential health risks [4,5,6]. 

Carbon dioxide, volatile organic compounds, biocontaminants, fungi, bacteria, and particulate matters are among the indoor air pollutants with damaging potential to human health listed by the European Federation of Allergy and Airway Diseases Patient Associations in their document [7]. Dust intake rates for children are estimated to be between 30 and 140 mg/day, whereas adults consume 2–30 mg/day [8,9]. 

According to [10,11], indoor dust can be described as tiny particles (≤100 μm) that settle in indoor spaces. These particles can come from various sources situated inside and outside the building. Particles with diameters smaller than 10 μm (PM_10_) can be inhaled, the coarse fractions being retained in the upper airways, and those particles with diameters less than 2.5 μm can reach the pulmonary system or enter the blood [12]. Particles with diameters from 1 µm to 20 µm are responsible for the apparition of asthma [13]. Tsubata et al. [14] indicate that dust particles with diameters less than 11 µm contain up to 90% of allergens. 

Research has indicated that indoor dust is a transporter for inorganic and organic contaminants, including heavy metals, pesticides, polychlorobiphenyls, and polycyclic aromatic hydrocarbons [5,6,15,16,17]. Indoor dust is a heterogeneous combination of particles that includes synthetic and natural fibers, hair, deposited atmospheric PM, biologically derived material (pollen, molds, bacteria, germs, animal fur, and dander), ash, skin particles, soot, and building and consumer product components [18]. Indoor dust typically contains about 35% outdoor soil, but this can vary widely based on factors like pets, shoe-wearing habits, and specific indoor settings. Indoor dust varies in organic content, typically ranging from 5% to 40%. Finer particles contain more organics, which are vital for absorbing pollutants. The fibrous particle content ranges from 9% to 89%, influenced by room type, furniture, and pet presence [19,20]. 

Pollutants enter the human body by inhalation, ingestion, and dermal contact [6,21,22,23,24]. According to [25], when inhaled, these toxic metals in dust can inflame, sensitize, and even scar the lungs and tissues because they are ubiquitous in the environment. Additionally, exposure to these metals may result in gastrointestinal issues, reproductive system problems, and nervous system disorders. Excessive exposure to Pb, Cd, Zn, and Cu is associated with the risk of cancer [26,27]. In this article, we analyze only the toxic metal found in indoor dust, whereas the dust microbiomes and metatranscriptomes have been studied in [28].

Bio-accessibility of heavy metals in indoor dust has been observed by physiologically based extraction tests or simplified bio-accessibility extraction tests based on the rationale that incidental oral ingestion is the main exposure pathway by which humans take in contaminants in indoor dust, especially for children [29,30,31].

Indoor air pollution poses a significant global health threat, contributing to around 4.5 million annual deaths worldwide. This pollution is responsible for a range of health issues, including pneumonia (12%), strokes (34%), ischemic heart diseases (26%), chronic obstructive pulmonary diseases (22%), and lung cancer (6%) [32,33]. Therefore, research on indoor air quality concluded that correct ventilation and proper cleaning [34,35] are necessary to avoid such health damage.

The International Agency for Research on Cancer (IARC) has classified Al, Co, Fe, Ni, and Zn as non-carcinogenic elements, whereas arsenic As, Cu, Cd, Cr, and Pb are classified as both carcinogenic and non-carcinogenic elements. The U.S. Environmental Protection Agency classified Cu, Cr, Ni, Zn, Cd, Mn, and Pb as environmental priority pollutants [36]. Moreover, it was shown that Cr, Cu, Ni, Zn, and Fe promote the exchange of electrons [34] and help the apparition of reactive oxygen species in the lungs [37].

On one hand, Cu is a micronutrient, a catalyzer of redox reactions, essential for the organism functioning. On the other hand, released in the atmosphere from anthropic (burning fossil fuel, solid waste management) and natural sources, it can attach to particulate matter and is transported long distances from its source [38].

Heavy metals like As, Cd, Cr, and Pb, which are widespread environmental pollutants, can cause health issues, including cancers, respiratory problems, cardiovascular diseases, nerve damage, and slow growth development [39,40,41,42]. 

Different particulate matter can also contain other elements like Ca, Li, and K transported by the wind, issued from the lithology of the place being studied. 

This article presents the analysis of indoor settled dust in Dubai, UAE, which holds significant importance since Dubai’s rapid urban development and construction activities are closely linked to indoor dust accumulation. Although studies on dust transportation and outdoor pollution (particularly with heavy metals) in different emirates from the UAE have been carried out [43,44,45,46,47], indoor pollution was less analyzed [28,48,49,50], with the emphasis on gaseous pollutants. Therefore, in this study, the composition of indoor settled dust from 20 important locations across Dubai is investigated using a complex approach involving a multivariate statistical analysis combined with different indices, two newly proposed here. It is shown that a correct conclusion on contamination cannot be drawn from a single index computation but from a combination of such indices, given that some elements present in high concentrations in the samples can have a significant influence on the classification. Moreover, comparisons of the clustering based on the row data and the quality indicators may highlight the differences between the sites where the samples were collected. 

## 2. Materials and Methods

### 2.1. Data Series

**Figure 1:**
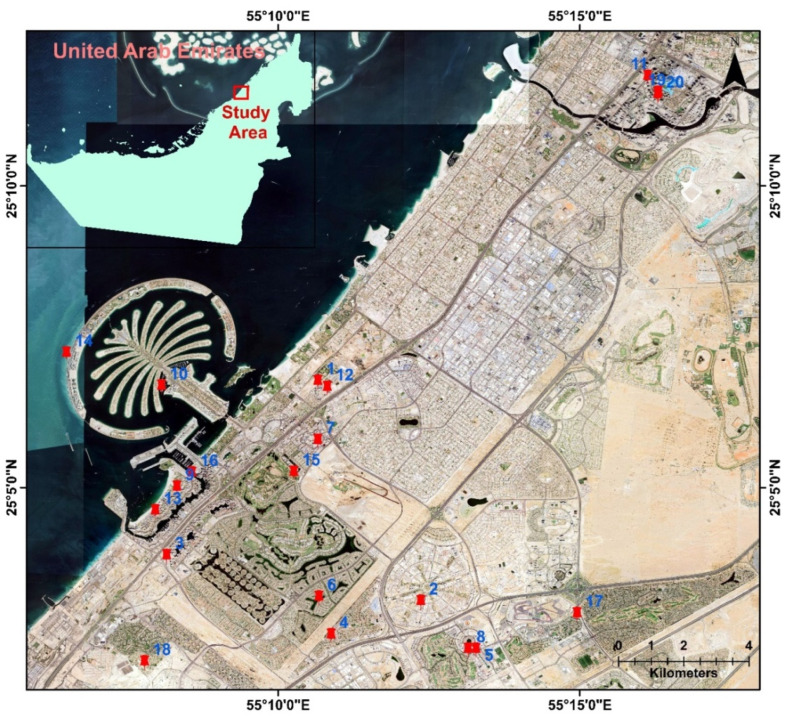
Study area location and sampling map. The red points and the numbers represent the sampling points and their IDs.

Dubai, located in the United Arab Emirates (UAE) is a remarkable city known for its unique blend of modernity and tradition. Situated on the Southeastern coast of the Arabian Peninsula (Figure 1), Dubai is one of the most prominent global cities, attracting tourists and business professionals from all over the world. It is bordered by the emirate of Sharjah to its north, while Abu Dhabi, the UAE’s capital, lies to the south. The climate of the study area is characteristic of the Arabian Peninsula, with hot and arid conditions prevailing throughout the year. Summers are exceedingly hot, with temperatures often exceeding 40 °C (104 °F). The city receives limited rainfall, and as a result, Dubai’s terrain is primarily desert, characterized by rolling dunes and sparse vegetation.

### 2.2. Sampling

Indoor-settled dust samples were collected using Dyson filters from twenty different locations in Dubai Emirates (Figure 1) including residential areas (Al Simmak Street, Bijada Blvd Street, Tulip Street), near heavy traffic junctions (Sheikh Zayed Highway), sports facilities (Sports City, Victory Heights), touristic areas (bars, restaurants), near water bodies (Dubai Marina) and commercial areas (markets, beauty lounges, butchers’ shops). Additionally, samples were also taken from specific buildings from Al Mustaqbal Street, Sheikh Mohammed Bin Rashid Blvd, a roundabout in Motor City, and near metro stations, offering a diverse range of environmental sources for analysis. The buildings’ characteristics differed, varying from the location, building materials, purpose, and maintenance. The dust samples were collected from undisturbed surfaces. Before sampling, the sites’ environmental conditions—temperature and humidity—were measured using a Graywolf Indoor Air Quality Meter (GrayWolf Sensing Solutions, LLC, Shelton, CT, USA) [51]. The measurements were performed when the atmospheric conditions were stable. The temperature inside was between 19 and 20 °C, and the relative humidity (RH%) was in the range of 40–45%. The coordinates of the observation sites were recorded using a South S750 Handheld GPS meter (Guangzhou, China) [52].

A Dyson V15 Detect vacuum machine with two heads (Gurugram, India) (separately collecting dust particles from rugs/carpets with a fluffy brush-bar and filter, and hard floors with a built-in laser light to observe the incoming material from the cleaning surface) was utilized. The Dyson vacuum has a HEPA post-motor filter that can trap particles with dimensions at least of 0.1 microns. Moreover, the dust particles are continuously counted and sized by a piezo-sensor [53]. 

A representative sampling strategy was adopted to collect the samples, which were transferred into re-sealable plastic bags by gently sweeping with fingers wearing powder-free nitrile gloves. They were safely packed and moved to the laboratory, where they were screened to remove any visible hair, soil, and grit. The samples were then air-dried for 48 h to avoid moisture in a well-protected area. All the results were reported based on dry weight.

### 2.3. Reagents, Standards and Laboratory Ware

In this research, all experiments were conducted using high-quality analytical reagent (AR) grade chemicals. We sourced the reference standard, check standard, and reagents from Sigma Aldrich (St. Louis, MO, USA). To create a 1:1 acid mixture, concentrated nitric acid (69% *v*/*v*) and hydrochloric acid (37% *v*/*v*) were combined. The water purity was ensured by using ultra-pure water with a chemical resistivity of 18.2 MΩ·cm from the Merck Millipore( Burlington, MA, USA) water purification system. For sample oxidation, 30% hydrogen peroxide was utilized. The equipment quality was maintained by using Class-A grade glassware for all the analyses. To eliminate potential contaminants, all items of glassware and plasticware were cleaned by washing them 5–6 times with ultra-pure water, and rinsing with 10% nitric acid, then drying them with an air drier. Later, sample digestion was carried out using the Mars-6 system from CEM in Matthews, NC, USA. Finally, ICP-OES analysis was conducted using OH, USA’s Perkin Elmer Avio 200 system. 

The sample digestion process followed the USEPA 3050B procedure (Washington, DC, USA) [54]. Initially, 0.2 g of each sample was weighed and placed into Teflon vessels for microwave-assisted digestion. Subsequently, 10 mL of a 1:1 HCl: HNO_3_ solution was added into the digestion vessel, thoroughly mixed with the sample slurry, and subjected to microwave digestion at 95 °C for 5 min. After digestion, the slurry was allowed to cool, and 5 mL of concentrated HNO_3_ was added. This mixture was then heated and refluxed at 95 °C for 5 min, followed by cooling and carefully adding 10% H_2_O_2_ for oxidation. The resulting solutions were transferred into 100 mL volumetric flasks, adjusted to the markup with water, and subsequently filtered using Whatman 41 filters (Maidstone, UK). The filtered solutions were subsequently subjected to analysis for heavy metals using an ICP-OES system, with eight replicate analyses conducted for each sample.

Quality control and assurance protocols were carefully observed throughout the sample preparation and analysis processes, encompassing laboratory blanks, check standards, and standard spiked samples. Laboratory blanks were prepared utilizing the same reagents employed for digestion but excluding the addition of dust samples. For all metals, the laboratory blank values were under the concentrations of metals in the target samples. The method detection limit (MDL) was calculated using the equation:

MDL = X + 2.896 × SD(1)

where X is the mean, SD is the standard deviation of blanks, and 2.896 is the value of the Student statistics at the significance level of 99%, and eight degrees of freedom. This equation has been used according to [55,56] because all the method blanks give either positive or negative numerical results. The MDL values ranged between 0.02 µg/kg (Cd) and 25.2 µg/kg (K). The metals recovery percentage (spiked and standard) was between 95% and 105%. The analytical precision for every metal of repeated analysis was determined by using the coefficient of variation, which was less than 3%.

### 2.4. Statistical Analysis

The first step in the analysis was the computation of the basic statistics—minimum (min), maximum (max), mean, median, standard deviation (std.dev.), coefficient of variation (CV), skewness coefficient, and kurtosis. The correlation matrix was determined to assess the correlation between the chemical elements in the dust.

After normalizing the data series, the set was submitted to clustering to group the 20 series recorded at different sites according to their common properties. For a better classification, the k-means algorithm [57] and hierarchical clustering [58] were used to cross-validate the results. Before performing the algorithms, the elbow [59] and silhouette [60] methods were utilized to choose the optimum number of clusters, k. 

Groups of series formed the output of the first technique, while that of the second one was a dendrogram that shows the series hierarchy and can be constructed by employing a certain distance, like the Euclidean one (utilized in this study). The degree of similarity between the elements in each group was estimated using different methods like “complete”, “average”, “ward.D2”, and “median”. The better-performing method was selected based on the highest value of the cophenetic correlation coefficient [61]. After clustering, bootstrapping was conducted to compute the average Jaccard measures, to ensure that the algorithm provided a good representation of the groups. A value of the Jaccard coefficient greater than 0.85 indicates a highly stable clustering, whereas one between 0.60 and 0.85 shows a stable grouping [62]. 

The next stage was to perform the Principal Component Analysis [63]. PCA is a multivariate statistical technique utilized for reducing the number of the observed parameters by replacing them with a smaller number of components, artificially created, called Principal Components (PC). The extracted PCs incorporate the highest part of the variance of raw parameters (usually above 80%) and are obtained as a linear combination of those parameters [64]. They can be considered independent factors that govern the development of a given process [65]. Among the criteria employed for the PC selection—Explained Variance Criterion [64,65], Catell Scree Plot [66], and Kaiser criterion [67]—the first two were utilized in this research.

The R 4.3.1 software (https://cran.r-project.org/, accessed on 15 October 2023) was the tool for performing the analysis.

### 2.5. Pollution Indices

To assess the pollution level or enrichment with the metals in the dust, the following indices were computed. They are:

For the metal *i*, $I_{geo}$ is calculated using the formula [68,69,70]:

(2) $$I_{geo}=log_{2}\left(C_{i}/\left(1.5CB_{i}\right)\right),$$

where $C_{i}\;$is the concentration of the *i-*th element in the dust and $CB_{i}$ is the value of the *i-*th element in the background.

The pollution index of the *j-*th element is given by [68]:

(3) $$PI_{j}=C_{j}/CB_{j}.$$

Values of *PI* in the intervals less than 1, 1–2, 2–3, 3–5, and greater than 5, respectively, indicate the contamination absence, low, moderate, strong, and very strong pollution, respectively.

The enrichment factor with the *j*-th element, $\;EF_{j},$ is defined by [68,69,70]:

(4) $$EF_{j}=[C_{j}/LV_{s}]/[CB_{j}/LV_{b}]$$

where $C_{j}$ is the concentration of the element *j* in the sample, $LV_{s}$ is the concentration of the reference element (generally Al, Ca, or Fe) in the sample, $CB_{j}$ is the reference concentration of *j*-th element in the background, and $LV_{b}$ is the concentration of the reference element in the background.

The background values utilized here are those from [71]. Same information can be found in [72] for different regions of the world.

Based on the value of the EF factor—less than 2, between 2 and 5, in the interval 5–20, between 20 and 40, or greater than 40—different classes of pollution are defined as deficient to minimal, moderate, significant, very high, and extremely high, respectively.

Aggregated indices can be computed from the individual ones to assess the contamination with multiple elements at a specific location. Two known indices were computed. The first one is *PLI*, defined by [73]:

(5) $$PLI={\left(\prod_{j=1}^{n}PI_{j}\right)}^{1/n}.$$

*PIs* of some elements (As, Ba, Co, Pb, in this case) are very low (of order 10^−2^), so they will artificially decrease the *PLI* value. Therefore, to have a correct evaluation of the contamination degree, the *PIs* corresponding to these elements were removed from the computation of the *PLI*, the resulting index, denoted by *PLI_d*, being also computed and compared with *PLI*.

The second one is the Nemerow index, calculated by [74]:

(6) $$PI_{Nem}=\sqrt{\left[{\bar{PI}}^{2}+PI_{max}^{2}\right]/2}$$

with

(7) $$PI_{max}=\max\left(PI_{1},\;\ldots ,\;PI_{n}\right)\;\mathrm{and}\;\bar{PI}=\left(\sum_{j=1}^{n}PI_{j}\right)/n.$$

Values less than 0.7, in the intervals 0.7–1, 1–2, 2–3, and higher than 3 are indicative of the absence of pollution, warning level, slight contamination, moderate pollution, and heavy contamination, respectively.

Two new indices are proposed, analogous to those used in water pollution assessment [75,76]. The first one, called in the following Combined Pollution Index (CPI), is defined by the formula:

(8) $$CPI=\frac{1}{n}\sum_{j=1}^{n}\left(C_{j}/CB_{j}\right).$$

We propose to keep as reference values those for $PI_{Nem}$.

The arithmetic weighted index is defined by:

(9) $$AQI=(\sum_{j=1}^{n}w_{j}Q_{j})/\left(\sum_{j=1}^{n}w_{j}\right),$$

with $w_{j}$ the weight associated with the quality index $Q_{j}$ of *i*th parameter,

(10) $$Q_{j}=100\times C_{j}/CB_{j},$$

(11) $$\;\;\;\;\;w_{j}=\frac{1}{CB_{j}}/\left(\sum_{j=1}^{n}\frac{1}{CB_{j}}\right).$$

The following classes are associated with the ranges (0–25)—unpolluted, (26–50)—warning level, (51–75)—slight pollution, (76–100)—moderate pollution, and (above 100)—heavy pollution.

## 3. Results and Discussion

Table 1 contains the basic statistics of the chemical elements series from the samples. The highest concentrations are those of Ca, K, Mg, Al, and Fe, and the lowest are those of Co, As, and Pb. Standard deviations (std.dev.) of most series of elements are high, indicating a high variation around the mean, but the variation coefficients are moderate. Only a few series present an accentuated skewness (Cr, Ba, Na, Mg), indicating a large variation range of the corresponding values.

The high concentrations of Cu, Mg, Fe, and Al in the dust might be explained by their existence in the natural rocks and anthropic activity. For example, there are 120 known occurrences of copper mineralization in the United Arab Emirates, situated in the mountainous region between Kalba and Dibba, or Wadi Hamm [77]. UAE is the seventh exporter of Mg in the world [78] and exported USD 53.6 M in iron ore in 2021 [79]. Moreover, it is the fifth aluminum-producing country in the world [80].

Studies indicate that indoor air quality is significantly affected by the outdoor air [81,82,83,84,85]. Kuo and Shen [83] found a similar increase in the concentrations of PM_2.5_ and PM_10_ in both indoor and outdoor air during a dust-storm event and interpreted the cause to be the extraction of outdoor air from their building’s ventilation system. The research of Ai and Mak [86] and Meier et al. [87] has shown that natural ventilation contributes to the deterioration of indoor air quality. Fisk [13] has found that the air in mechanically ventilated buildings enters from a small number of intakes so that the indoor air quality is significantly affected by the intakes’ neighboring sources situated outdoors. An extended review of the research on the correlation between indoor and outdoor air quality was performed in [88]. Therefore, in the case study, the high concentration of Mg, Fe, and Al from the indoor dust (highly correlated to that from outdoors), originates from the soil dust composition of a desert area, but one cannot ignore the contribution from industrial activities. The above-mentioned mining operations can introduce additional concentrations of minerals like Mg, Fe, and Al into the environment, and dust storms, frequent in the region, can transport these minerals over broader areas. To assess the minerals’ origin in the indoor dust, samples should be analyzed in future studies.

**Figure 2:**
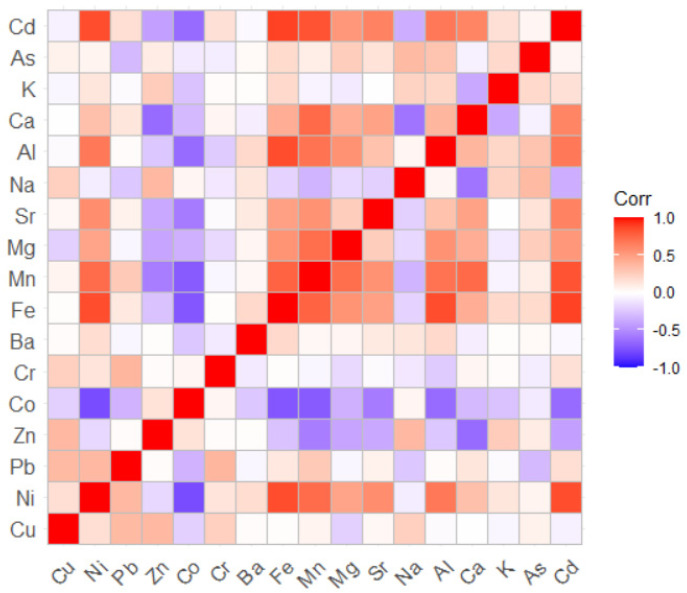
The correlation matrix. The higher the positive correlation, the more intense the nuance of red. The higher the negative correlation, the more intense the nuance of blue is. The nuances of light yellow, light orange, and indigo indicate a low or inexistent correlation.

Figure 2 presents the correlation matrix. The colors closer to red indicate a higher positive correlation between elements, and those closer to dark blue show a higher negative correlation.

Table 2 contains the p-values associated with the correlations between the chemical elements in the dust samples. The p-values less than 0.05 indicate a correlation between the elements. The lower the p-value, the higher the correlation is. Significant correlations are between the pairs Co–Ni, Fe–Ni, Mn–Ni, Mn–Mg, Mn–Sr, Mn–Al, Mn–Cd, Zn–Mg, Zn–Ca, Co–Fe, Co–Mn, Co–Sr, Co–Al, Co–Cd, Fe–Mn, Fe–Mg, Fe–Sr, Fe–Al, Fe–Cd, etc. This means that significant correlations are found between the metals in the dust resulting mainly from industrial activities and transported for long distances by the wind.

**Figure 3:**
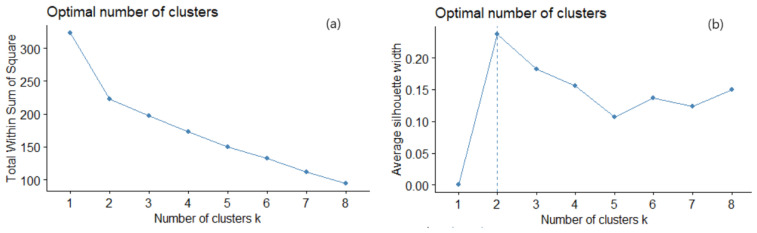
(**a**) Elbow and (**b**) silhouette methods for selecting the number of clusters.

The optimal number of clusters, *k*, determined by the elbow and silhouette (Figure 3) was two (Figure 3).

After bootstrapping, the calculated average Jaccard values were 0.983 and 0.980, and the corresponding instabilities were 0.005 and 0.014. So, the groups found are highly stable. The first cluster contains the samples collected mainly from Dubai downtown, Burj Khalifa, near crowded zones, and in the vicinity of sandy zones. The second one is formed mainly by locations situated near the seafront, in green zones, and residential areas. The sampling series from the first cluster mainly contains the highest Pb, Zn, and Co concentrations and the lowest concentrations of Ni, Mn, and Mg.

**Figure 4:**
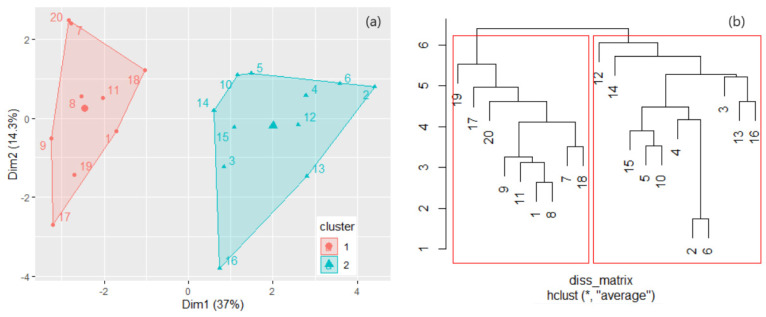
(**a**) The clusters found by k-means with *k* = 2; (**b**) Dendrogram in the hierarchical clustering.

The clusters obtained by the k-means algorithm (*k* = 2) are presented in Figure 4a. The dissimilarities between the elements in two clusters, in the hierarchical clustering, were assessed by different methods, among which “average” best performed in terms of cophenetic correlation coefficient (which was the highest compared to those of “complete”, “average”, “ward.D2”, and “median” procedures). In this method, all pairwise dissimilarities between the elements in two clusters were computed, and the distance between clusters was calculated by averaging these dissimilarities.

After bootstrapping, the obtained average Jaccard values (instabilities) were 0.828 (0.146) and 0.826 (0.172), showing that the clusters are stable. The dendrogram resulting from the hierarchical clustering is displayed in Figure 4b. Comparing Figure 4a,b, one may observe that both methods provided the same clusters.

PCA found 17 PCs, corresponding to the same number of chemical elements. However, Table 3 provides the computation results of only five PCs, including the proportion of the variance explained by each component, the cumulative proportion, and the standard deviation. The first two (three) PCs explain 80.90% (89.5%) of the variance. So, PC1 explains more than two-thirds of the information provided by the 17 variables, whereas PC2 and PC3 explain, respectively, 11.53% and 8.58% of the total variance.

**Figure 5:**
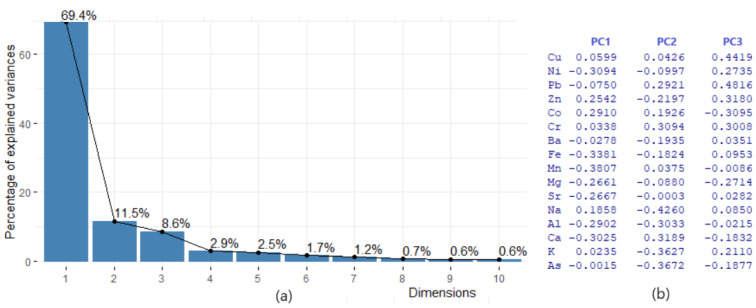
(**a**) The screen plot and (**b**) loading table.

The cumulative proportion of PC1–PC3 is about 89.46% of the total variance. So, PC1–PC3 (or even only PC1 and PC2) can accurately represent the data set. The screen plot that reflects this information is shown in Figure 5a.

**Figure 6:**
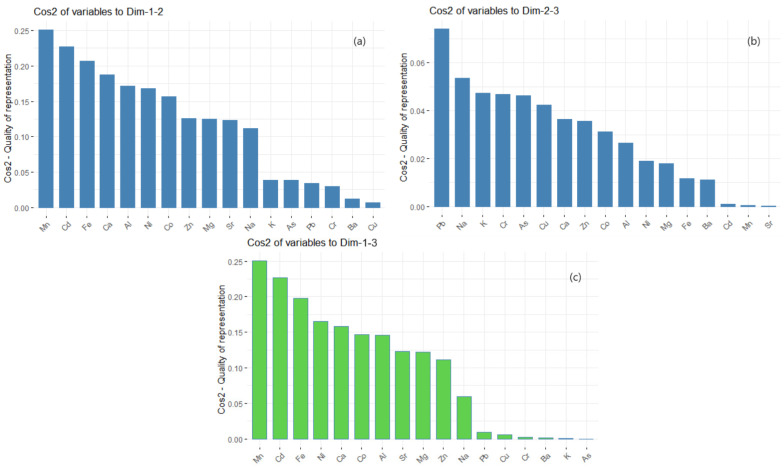
(**a**) Cos2 of variables to Dim 1–2; (**b**) Cos2 of variables to Dim 2–3; (**c**) Cos2 of variables to Dim 1–3.

**Figure 7:**
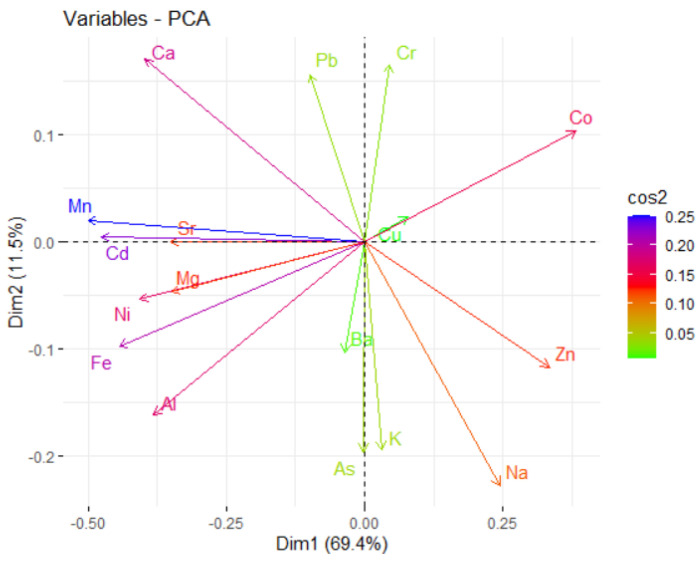
Variables—PCA.

The PC score (factor loading) of each variable in a PC indicates the processes controlling the variability of the data [89]. The loading table (Figure 5b) shows that the first principal component has high positive values for Co and Na. The values for Mg, Cd, Ca, and Ni are negative. This suggests that sites with a component of Co and Na in the dust are in excess. In PC2, Ca, Cr, and Pb are in excess, while the negative contributions come from Na, As, K, and Al. The highest contributions on PC3 are of Pb, Cu, Zn, Cr, and Ni. Therefore, the main contributions are those of Cr, Cu, Zn, Pb, Ni, and As, resulting mainly from human activities (transportation and industry). The variables’ quality representation on the factors map (cos2 representation) is shown in Figure 6. The better the representation, the higher the cos2 is. So, the groups (Mn, Cd, Fe, and Ca), (Pb, Na, K, and Cr), and (Mn, Cd, Fe, and Ni) are, respectively, the best represented on the first three PCs. The variables’ contributions in different dimensions are also represented in Figure 7.

The highest absolute values on PC1 are represented in nuances of blue. They are Mn, Cd, Fe, and Ca. Note that Mn, Cd, and Sr are grouped, indicating their correlation. The same remark stands for Mg, Ni, and Fe. Co is negatively correlated with Fe and Al; the same remark for Na and Pb, etc.

The contamination levels with respect to the $I_{geo}$ values from the literature and the degree of contamination at the studied sites are presented in Table 4. The elements with significant impacts at all sites are Fe, Mg, Ca, and K.

With respect to PI, no pollution with Cu, Ni, Pb, Co, Cr, Ba, Mn, Sr, As, or Cd was found. Low pollution with Zn was found at the sites 1, 4, 5, 7–12, 15, 18–20, Al—9, 17, and 20. Moderate pollution was that with Fe (at 7, 9, 17), Mg (at 17), and Al (at 1, 3, 7, 8, 10–16, 18). Strong pollution was noticed with Fe (at 1, 3, 5, 8, 10–16, 18–20), Mg (at 7–9, 11, 19, and 20), and Al (at 2 and 4–6). Very strong pollution was registered with Fe (at 2 and 4) and Mg (1–6, 10, 12–16, and 17). The PI for Na falls between 1 and 2, at sites 1, 5–7, and 18, and between 2 and 3 at 20. PIs for Ca and K are greater than 5 at all sites.

Based on the EF computed with respect to Al, moderate enrichment was seen for Fe (at sites 8, 9, and 19), with Mg (at 1, 2, 4, 6, 8–10, 12–16, and 18–20), and K (at 1–6 and 12–17), whereas significant enrichment was determined only with Mg at site 3, and K (at 7–11, and 18–20).

The EF calculated with respect to Ca shows that all the sites are in the same category of deficient to minimum enrichment. EF computed with respect to Ca indicates a moderate enrichment in K (at all sites but 6, 7, and 18) and Mg (at site 3). Significant enrichment in K was determined at sites 7 and 18 and in Ca at all sites.

**Figure 8:**
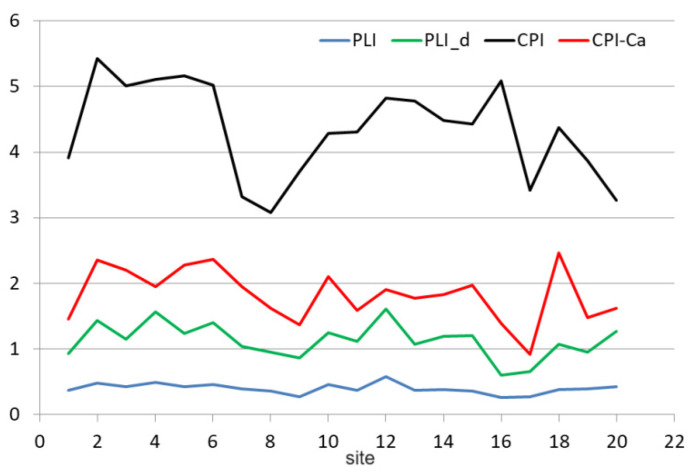
CPI and CPI-Ca.

PLI values are between 0.26 and 0.58, so less than 1, proving a variation between perfection (indicated by a value of 0) and baseline (shown by a value of 1). Since the PIs corresponding to Co, As, Cd, and Pb are under 0.03, they contribute to the decrease in *PLI* values. Removing these elements from computation, denoted by *PLI_d*, produced values from 0.60 to 1.61 (Figure 8). *PLI_d* is more than two times higher than *PLI*.

*PLI_d* indicates a variation between perfection and baseline (0 < *PLI* < 1) for sites 1, 8, 9, 16, 17, and 19, and there is a progressive deterioration of the air quality (1 < *PLI* < 1.61) for all sites but those already mentioned. Locations 2, 4, 6, 12, and 20 have the highest *PLI* and *PLI_d*, and so the biggest contamination, as shown in Figure 8. All but site 20 belong to the second cluster in Figure 4a. 

Taking into account all *PIs*, the Nemerow pollution index obtained values between 18.02 and 45.56, indicating high contamination at all locations. Removing the *PI* for Ca, the values of the index, denoted $PI_{Nem-Ca}$, varied in the interval 3.67–16.89. Notice the essential influence of the very high PIs on the values of the Nemerow index.

All values of the *CPI* index (Figure 8) were between 3.09 and 5.43, indicating heavy pollution.

Since Ca is an element that has mainly a natural origin, and we did not find essential evidence of another origin in the region, removing it from the *CPI* computation (and denoting the new index by *CPI-Ca*), the variation in *CPI-Ca* was in the interval 0.92–2.46. Therefore, the pollution level from site 17 is graded warning, locations 2, 3, 5, 6, 10, and 18 are moderately polluted, and the rest are slightly contaminated.

The $PI_{Nem}$ and *CPI* are influenced by the highest values of the ratio $C_{j}/CB_{j},\;$in contrast with the *PLI*, whose values are more related to the elements’ lowest concentrations.

**Figure 9:**
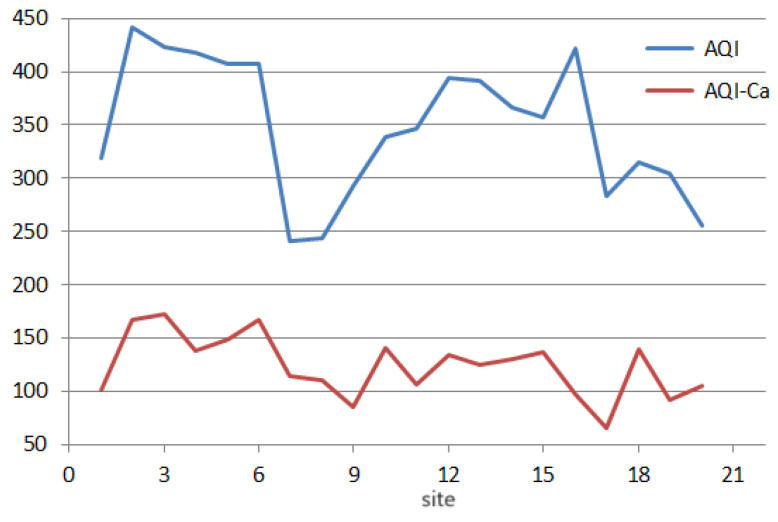
AQI and AQI-Ca.

Computation of the *AQI* (Figure 9) taking into account all elements (case a) or without calcium (case b—*AQI-Ca*) resulted in (a) heavy pollution at all sites, respectively, and (b) slight pollution at site 17 and moderate contamination at sites 9, 16, 19. Three of these locations are situated in the same cluster from Figure 4a. The shapes of the *CPI* and *AQI* indices charts are similar. A significant decrement in their values is noticed when Ca is removed from computation.

**Figure 10:**
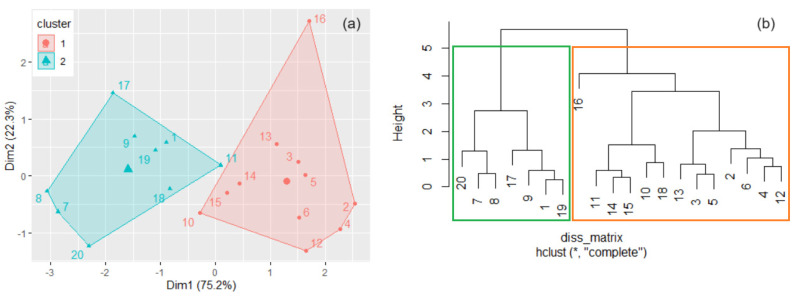
(**a**). The clusters found by k-means with *k* = 2; (**b**) Dendrogram from the hierarchical clustering for Set2.

As mentioned above, the PIs computed for four elements were under 0.01, and they were removed from the computation of the *PLI*, leading to obtaining *PLI_d*. For consistency, we removed these elements from the initial data set (let us denote it Set1), obtaining set S2 that contained only 13 series of elements. The same analysis as that presented above has been performed for Set2. We only summarize the findings:

➢ The k-means algorithm and hierarchical clustering provided the same clusters and dendrogram as in Figure 4, indicating that the removed series does not have a significant importance to lead to a difference between the sites.
➢ The Nemerow indices computed with Set2 are the same (up to the third decimal) as those computed using Set1, while the *CPI* and AQI have higher values for Set2.
➢ The k-means algorithm performed on the series of indices obtained from Set2 provided the same clusters as in Figure 4a—see Figure 10a.
➢ The hierarchical clustering performed on the series of indices obtained from Set2 provided a cluster containing the series 1, 7–9, 11, 17, 19, and 20 which are also in the left-hand-side cluster from Figure 4b.

**Figure 11:**
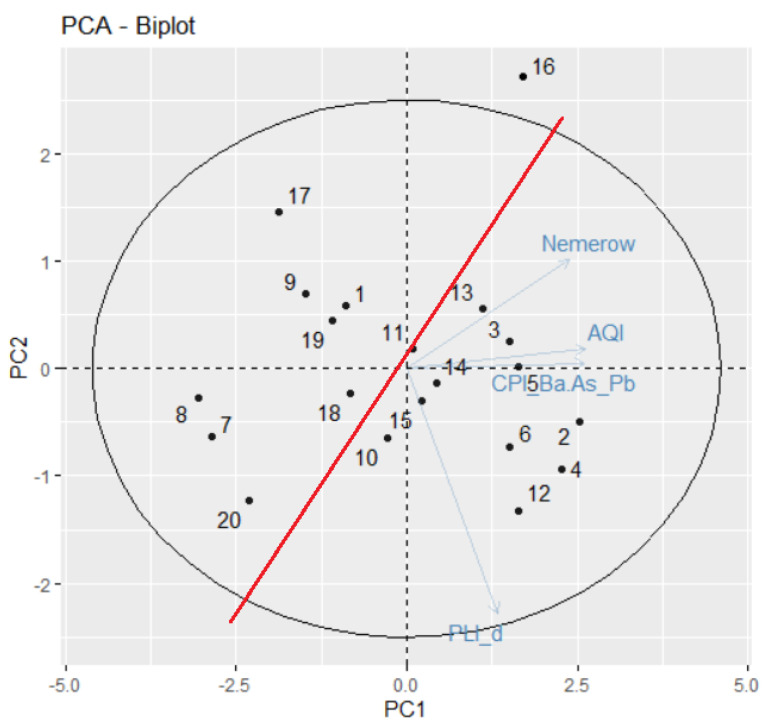
Biplot in PCA for Set2.

Figure 11, the biplot obtained performing the PCA for Set2 indicates the positions of the sites in the first cluster from the dendrogram—all grouped at the left-hand side of the biplot, with negative components on PC2. A clear separation line (the red one) can be drawn between the two clusters. The locations from the first cluster are in tourist areas—Dubai Marina, Burj Khalifa, and Dubai Sport City. Site 16 is situated on Dubai Marina Promenade, near the water, in a restricted area for cars, a zone with the lowest recorded concentrations of the study metals. This particular situation is emphasized by its position on the biplot.

We should remember that, generally, the perfect superposition of the clusters determined by both methods is a particular situation given the various mathematical backgrounds on which the algorithms rely. In the case of homogenous sets, it is expected (which is not the case in this study).

Performing the algorithm to determine the clustering by elements, two clusters were obtained, one with only two elements As and Cd (when working with Set1), and Ca and K (when working with Set2). This situation pointed out the elements with the lowest and highest concentrations, respectively, the last ones requiring attention.

## 4. Conclusions

This article analyzed the degree of enrichment with metals of the dust collected indoors at different locations in Dubai, using multivariate statistics and pollution indices. The study fills a gap in the knowledge concerning indoor pollution due to dust in a region where frequent dust storms appear.

It was shown that the highest enrichment factors (for Ca, Cu, Mg, and Fe) are the consequence of the soil lithology and industrial activities (especially mining), dust being transported for long distances from the emission places during dust storms.

We proposed two new pollution indices—CPI and AWI—and used them for assessing the contamination at the observation places. We classified the sites based on the set formed by the PLI, CPI, AWI, and the Nemerow index and compared it with that built by row data series. It was found that two sites fall into different clusters resulting from these classifications.

Another finding that opens a research direction is using different groups of data sets for classifications in practical applications. It was shown that for the clusters built when eliminating the elements with the lowest concentrations (much under the warning limits) from the data set, the obtained classifications are more realistic.

Employing different clustering algorithms on the raw data series and the pollution indices series, and the use of stability criteria, are important for finding the most similar series in the data set (those that are found all the time together in the same cluster).

In a future study, we intend to present a methodology that will come to cross-validate the clustering findings, using supplementary selection criteria and decision trees.

## Figures and Tables

**Table 1:** Basic statistics of the series of elements from the dust samples [mg/kg].

	**Cu**	**Ni**	**Pb**	**Zn**	**Co**	**Cr**	**Ba**	**Fe**	**Mn**
min	3.04	29.85	0.05	25.81	0.16	19.14	28.96	568.36	66.38
mean	94.30	52.14	4.62	247.08	1.85	56.96	85.17	997.28	126.58
max	309.58	93.50	28.82	397.11	3.62	298.47	309.94	1572.14	186.24
median	53.54	47.10	2.37	255.84	1.91	33.27	74.36	979.05	133.08
Std.dev.	97.74	18.80	6.72	92.52	0.95	63.60	55.28	263.67	43.47
CV	1.04	0.36	1.46	0.37	0.52	1.12	0.65	0.26	0.34
Skewness coef.	1.53	0.72	2.86	−0.57	0.04	3.06	3.54	0.40	−0.04
Kurtosis	0.65	−0.57	8.47	0.10	−0.76	10.31	14.39	−0.03	−1.71
	**Mg**	**Sr**	**Na**	**Al**	**Ca**	**K**	**As**	**Cd**	
min	834.32	11.44	188.15	349.33	8033.17	3918.61	0.64	6.26	
mean	1876.22	47.50	561.39	1033.78	14,170.02	9159.12	3.89	6.73	
max	4972.55	120.35	1606.82	1883.38	20,421.29	17,984.38	5.61	7.45	
median	1843.27	44.64	493.99	965.54	14,436.28	8661.69	4.26	6.68	
Std.dev.	928.09	23.21	295.98	391.79	3085.02	2873.00	1.41	0.32	
CV	0.49	0.49	0.53	0.38	0.22	0.31	0.36	0.05	
Skewness coef	1.86	1.43	2.43	0.70	−0.36	1.28	−0.85	0.46	
Kurtosis	5.10	3.52	7.29	0.60	0.04	3.52	−0.01	−0.43	

**Table 2:** *p*-values related to the correlation coefficients of the elements found in the dust. The shaded cells, containing *p*-values less than 0.05 (the level of significance), indicate the existence of a significant correlation between the elements from the line and columns that intersect at that cell.

	Cu	Ni	Pb	Zn	Co	Cr	Ba	Fe	Mn	Mg	Sr	Na	Al	Ca	K	As
**Ni**	0.465															
**Pb**	0.118	0.104														
**Zn**	0.105	0.484	0.945													
**Co**	0.386	0.000	0.153	0.537												
**Cr**	0.280	0.546	0.101	0.930	0.825											
**Ba**	0.936	0.446	0.869	0.966	0.300	0.705										
**Fe**	0.961	0.000	0.605	0.267	0.000	0.955	0.400									
**Mn**	0.817	0.000	0.226	0.011	0.000	0.862	0.881	0.000								
**Mg**	0.391	0.035	0.867	0.093	0.146	0.495	0.833	0.012	0.000							
**Sr**	0.882	0.007	0.763	0.106	0.009	0.924	0.637	0.027	0.011	0.272						
**Na**	0.285	0.734	0.314	0.109	0.822	0.676	0.590	0.433	0.164	0.469	0.401					
**Al**	0.937	0.001	0.949	0.302	0.002	0.351	0.387	0.000	0.001	0.011	0.168	0.838				
**Ca**	0.963	0.159	0.587	0.002	0.202	0.824	0.783	0.068	0.000	0.066	0.030	0.005	0.100			
**K**	0.880	0.596	0.924	0.257	0.276	0.923	0.957	0.396	0.847	0.717	0.978	0.336	0.372	0.108		
**As**	0.784	0.816	0.200	0.671	0.705	0.739	0.909	0.416	0.700	0.265	0.516	0.116	0.188	0.804	0.389	
**Cd**	0.816	0.000	0.461	0.076	0.002	0.493	0.890	0.000	0.000	0.016	0.003	0.125	0.001	0.004	0.493	0.829

**Table 3:** PCA.

	PC1	PC2	PC3	PC4	PC5
Standard deviation	1.314	0.536	0.463	0.271	0.249
Proportion of Variance	0.6935	0.1152	0.0858	0.0294	0.0249
Cumulative Proportion	0.6935	0.8087	0.8946	0.9241	0.9489

**Table 4:** *I_geo_* values, corresponding contamination levels, and the sites included in each class [90].

Igeo Class	Igeo Value	Contamination Level	Contamination Level at the Study Sites
0	*I_geo_* ≤ 0	Uncontaminated	Cu, Ni, Pb, Co, Cr, Ba, Sr, Mn, Sa Cd—all sitesZn: 1–6, 12–14, 16–19; Na: 1–6, 8–19; Al: 9–19, As, Cd
1	0 *< I_geo_ <* 1	Uncontaminated/Moderately contaminated	Zn: 7–11, 15, 20; Na: 7, 20; Fe: 7, 9, 17; Mg: 17; Al: 1, 3, 7, 8, 10–18, 20
2	1 *≤ I_geo_ <* 2	Moderately contaminated	Fe: 1, 3–5, 8, 10–16, 18–20; Mg: 1, 7–9, 11, 18–20; Al: 2, 4–6; K: 17
3	2 *≤ I_geo_* < 3	Moderately/Strongly contaminated	Fe: 2, 6; Mg: 2, 4–6, 10, 12–16; K: 1, 3, 4, 8, 9, 11–13, 15, 20
4	3 ≤ *I_geo_* < 4	Strongly contaminated	Mg: 3; K: 2, 5, 7, 9, 15, 18, 19;
5	4 ≤ *I_geo_* < 5	Strongly/Extremely contaminated	Ca: 1, 6–11, 14, 15, 17–20
6	*I_geo_ ≥* 5	Extremely contaminated	Ca: 2–5, 12, 13, 16

## Data Availability

Data will be available on request from the author.
